# Long-Lasting Insecticidal Nets Are Synergistic with Mass Drug Administration for Interruption of Lymphatic Filariasis Transmission in Nigeria

**DOI:** 10.1371/journal.pntd.0002508

**Published:** 2013-10-31

**Authors:** Abel Eigege, Alphonsus Kal, Emmanuel Miri, Adamu Sallau, John Umaru, Hayward Mafuyai, Yohanna S. Chuwang, Goshit Danjuma, Jacob Danboyi, Solomon E. Adelamo, Bulus S. Mancha, Bridget Okoeguale, Amy E. Patterson, Lindsay Rakers, Frank O. Richards

**Affiliations:** 1 The Carter Center, Jos, Plateau State, Nigeria; 2 University of Jos, Jos, Plateau State, Nigeria; 3 Plateau State Ministry of Health, Jos, Plateau State, Nigeria; 4 Nasarawa State Ministry of Health, Lafia, Nasarawa State, Nigeria; 5 Nigeria, Federal Ministry of Health, Abuja, Nigeria; 6 The Carter Center, Atlanta, Georgia, United States of America; Liverpool School of Tropical Medicine, United Kingdom

## Abstract

In central Nigeria *Anopheles* mosquitoes transmit malaria and lymphatic filariasis (LF). The strategy used for interrupting LF transmission in this area is annual mass drug administration (MDA) with albendazole and ivermectin, but after 8 years of MDA, entomological evaluations in sentinel villages showed continued low-grade mosquito infection rates of 0.32%. After long-lasting insecticidal net (LLIN) distribution by the national malaria program in late 2010, however, we were no longer able to detect infected vectors over a 24-month period. This is evidence that LLINs are synergistic with MDA in interrupting LF transmission.

## Author Summary

In Plateau and Nasarawa states in central Nigeria, 4 million persons are threatened by a mosquito-transmitted parasitic disease called lymphatic filariasis (LF). LF can lead to elephantiasis, a crippling condition in which the limbs and genitals often are grotesquely swollen or enlarged. In communities afflicted by this disease, as many as 10% can be affected with swollen limbs, and 50% of men can suffer from swollen genitals. These conditions have a devastating effect on the quality of life of victims, impacting them not only physically but also emotionally and economically. Through health education and community-delivered mass drug administration (MDA) with donated medicines, the Nigerian Ministry of Health and its Carter Center partners have been trying to stop mosquitoes from transmitting LF. LF transmission, as measured by mosquito dissections, dropped dramatically after 8 years of annual MDA. However, it was not until the malaria program distributed long-lasting insecticidal nets in 2010 that the LF parasite no longer appeared in mosquito dissections. No LF infected mosquito was found over a 24-month long surveillance period following long-lasting insecticidal net distribution. The study concluded that MDA and long-lasting insecticidal nets work together to halt the transmission of LF.

## Introduction

Richards et al. [1], in a paper published in this journal in October 2011, reported on the results of efforts to stop transmission of lymphatic filariasis (LF) during the period 1998–2009 in central Nigeria (Plateau and Nasarawa states). LF in this area is caused by *Wuchereria bancrofti*, the vector being *Anopheles gambiae* s.l. and *An. funestus*. The strategy used was the World Health Organization (WHO) approved approach of providing the combination of ivermectin and albendazole in mass drug administration (MDA) programs, with health education, reaching ≥85% of the treatment eligible population of 3.7 million.

To determine impact on transmission, we monitored three LF infection parameters (nocturnal microfilaremia (mf), LF antigenemia, and mosquito larval infection) in 10 sentinel villages (SVs). In our last report, after SVs had been treated for 7–10 years, mf had decreased by 83% from baseline (from 4.9% to 0.8%); antigenemia by 67% (from 21.6% to 7.2%); mosquito infection rate (all larval stages) by 86% (from 3.1% to 0.4%); and mosquito infectivity rate (L_3_ stages) by 76% (from 1.3% to 0.3%). We expressed our concern about continued observations of larval stages of the parasite (especially the infective L_3_ stages) in mosquito dissections.

In late 2010 (Plateau state) and early 2011 (Nasarawa state), the national malaria program and its partners (including The Carter Center) successfully accomplished community wide long-lasting insecticidal net (LLIN) distribution in the two state area. MDA continued during the years 2011 and 2012. We report herein new entomological findings that document the disappearance of dissection detectible *W. bancrofti* larvae in mosquitoes captured in the SVs during a 2-year period after LLIN distribution, strongly implying synergy between LLIN and MDA in this area.

## Methods

### Ethics Statement

The LF and malaria programs are programs of the Federal Ministry of Health initiative. The entomological monitoring procedures were approved by the Plateau and Nasarawa state Ministries of Health and by the Emory University Institutional Review Board (protocol nos. 609-97, 153-2001, and 435-2003). Informed consent was first given by the village chief and his council. Then informed consent was obtained from residents of the houses being monitored by pyrethrum knock-down (PK). The team obtained informed consent by reading a previously prepared statement with a description of the purpose of the program, and risks and benefits of the PK. Oral consent was approved by the Emory IRB because literacy rates are very low in the rural SV areas. Consent was written when residents were literate. The statement texts were approved by the IRB. Consent was documented on individual forms, and in the case of oral consent, the responses to the questions were ticked off by the team leader.

**Figure 1:**
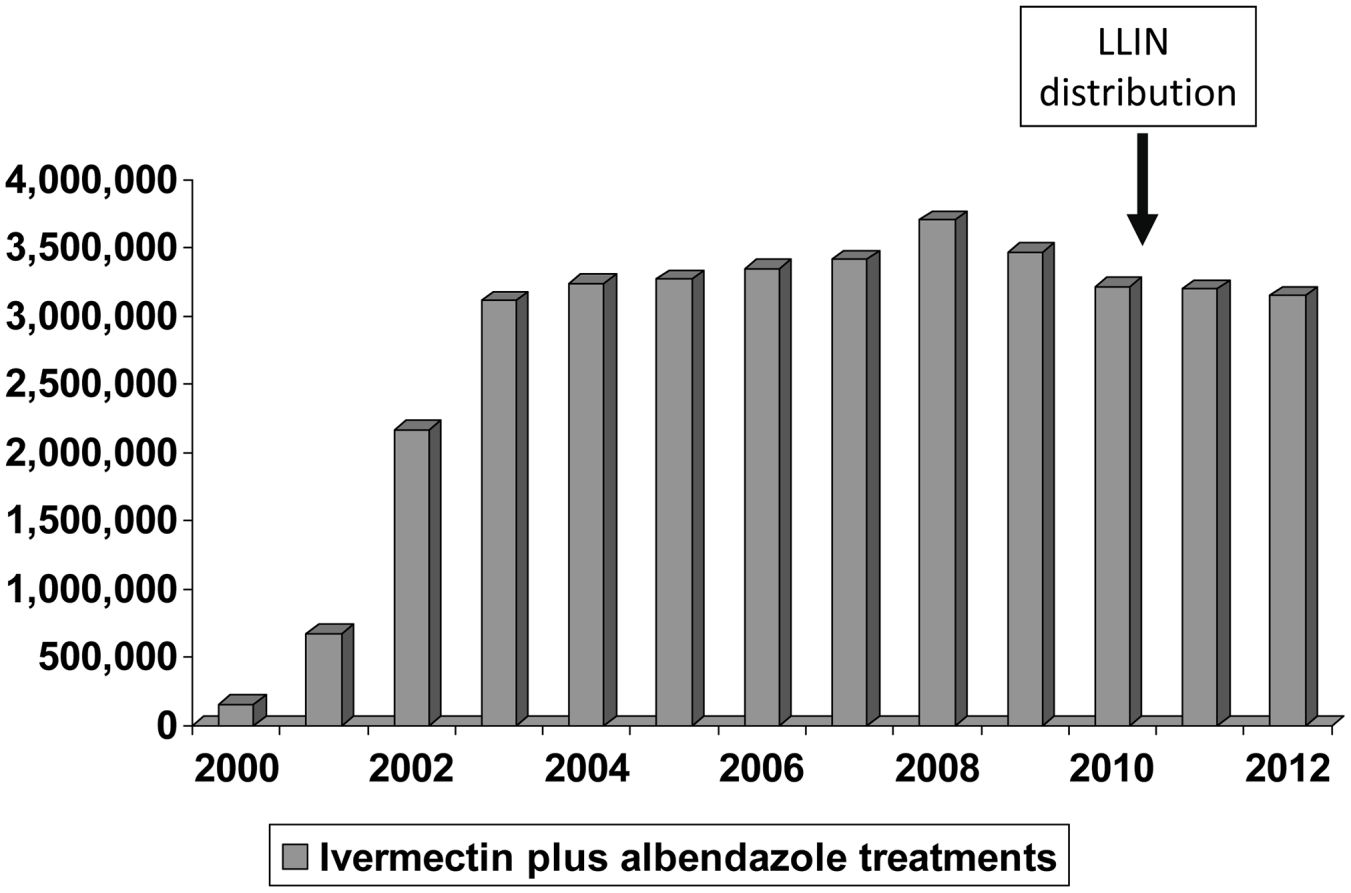
13 years of mass drug administration for LF in Plateau and Nasarawa states, Nigeria, 2000–2012 (n = 36,119,921).

The MDA activities, location of the 10 SVs, and the entomological monitoring were unchanged from that previously described in detail [1]. Briefly, the entomological surveys were conducted every 2 months in each SV. Indoor resting mosquitoes were collected in the morning using the PK technique. Dissections were performed on the day of collection and a trained microscopist noted the presence or absence of larval stages (L_1_–L_3_). Only results from *Anopheles* mosquitoes are reported. Infected mosquitoes were defined as having any *W. bancrofti* larval stage (L_1_, L_2_, or L_3_). Infective mosquitoes were defined as those containing L_3_. SV results from the six outings of the year were summed; at least 100 mosquitoes needed to be dissected in a year for that SV result (of that year) to be included in the analysis. ‘Baseline’ mosquito infection rates were aggregate results from ‘pretreatment’ and the first 2 years of ivermectin plus albendazole MDA. The MDA program took 4 years to scale up to full geographic coverage (Figure 1). As a result, different SVs had different MDA exposure histories. The aggregate entomological results from the last 2 years of MDA alone (calendar years 2009 and 2010) were from the SVs when they had received 8–11 MDA rounds. Aggregated dissection results from the same SVs 1–2 years after LLIN were distributed (calendar years 2011 and 2012) represented combined MDA (rounds 10–13) supplemented by universal LLIN coverage/use.

### LLIN Distribution Process

As part of the nationwide scale-up of LLIN coverage in Nigeria, 1,451,558 LLINs were distribution in Plateau state in December 2010 and 842,342 in Nasarawa state in January 2011, through the combined efforts of many partners including The Carter Center. LLIN distribution linked with the LF program has long been a Carter Center interest in the area [2]. The 2010–2011 mass campaigns employed a two-nets-per-household distribution strategy, and were accompanied by advocacy and health education activities in order to increase participation in the campaigns and to achieve the target of 80% net use. Each household was provided with a (unique serial numbered) voucher entitling the household members to receive two nets. These vouchers were exchanged for nets at local distribution points on specified dates.

## Results

### MDA and LLIN Distribution

Figure 1 shows the scale up of the MDA program and the number of treatments provided by year. The arrows indicate when the 2.29 million LLINs were distributed in 2010 and 2011. Voucher redemption rates of 98.9% and 97.9% (for Plateau and Nasarawa, respectively) were calculated by matching the returned vouchers to the serial numbers on the voucher stubs. Total MDA treatments provided in the two state area were essentially unchanged in the years after the LLIN distribution compared to prior to LLIN distribution.

### Mosquito Dissection Results

**Figure 2:**
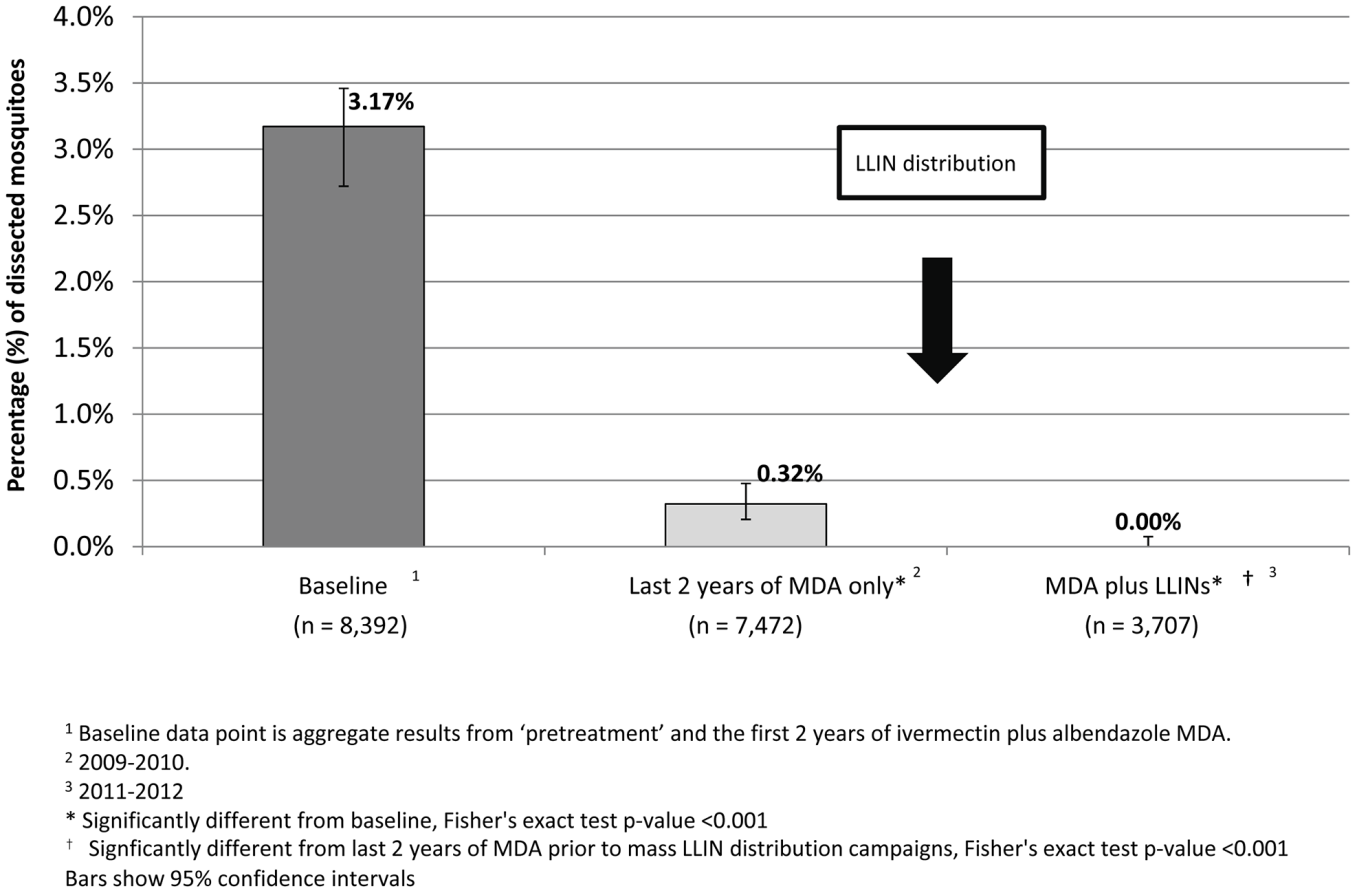
Mosquito lymphatic filariasis infection rates (all larval stages) in Plateau and Nasarawa state sentinel sites.

Figure 2 shows the mosquito infection results from 19,571 dissections. MDA alone decreased infection rates by over 90% from a baseline of 3.17% to 0.32% in the 2 years prior to LLIN distribution. After distribution of LLIN, mosquito collections (abundance) decreased by almost 49.6% and no infected mosquitoes were found. All findings were highly statistically significant (p<0.001).

Entomological monitoring in the SVs detected L_3_ in mosquitoes every year during the MDA-alone intervention. Infective rates among mosquitoes were 1.3% at baseline and 0.2% during the last years of MDA alone (2009–2010). However, after LLIN were distributed, no L_3_ were detected in mosquitoes during 2011 and 2012 entomological monitoring.

## Discussion

In our last report [1] we expressed our concern that even after 10 years of MDA low-grade mosquito infection (including L_3_) persisted. The importance of this entomological finding was unclear. Pedersen et al. [3] determined that the *Anopheles* transmission ‘breakpoint’ would be at an infection rate below 0.65%, and the program had achieved this threshold using MDA alone (Figure 2). Nonetheless, we welcomed the additional intervention of universal distribution of LLIN (with of goal of providing two LLIN per household) throughout Plateau and Nasarawa states, provided by the national malaria program and its partners. The 2010/2011 LLIN distribution had a significant impact on our entomological findings in the SVs. These findings mirror those reported in an LLIN-only approach utilized in south-east Nigeria, where the program area experienced a statistically significant decrease in LF infection and infectivity [4]. Entomologically, it became evident in the SVs that LF transmission was completely interrupted after 2010. The reductions in mosquito abundance and infection rates are evidence that the national malaria program will accelerate the elimination of LF.

A weakness of this study is that there were no control SVs where LLIN were not distributed to demonstrate that in those villages LF infection would still have been observed in mosquitoes where the only intervention was MDA.

We conclude that LLINs are synergistic with ivermectin and albendazole MDA. Our observations are an important addition to the published literature on the subject of LLIN MDA synergy [5], [6], [7], [8], [9], [10], [11]. We recommend the LF community become actively involved in assisting the malaria efforts in Africa and use community-level MDA mechanisms to maximize and sustain community-wide LLIN delivery and use.

## Supporting Information

Checklist S1 **STROBE checklist.**
(PDF)
